# Depressive Symptoms in Older Adults via Multimodal Markers on Magnetic Resonance Imaging: A Literature Review

**DOI:** 10.14283/jarlife.2023.2

**Published:** 2023-04-19

**Authors:** M. Montoya-Martinez, C. Arbus, K. Virecoulon Giudici

**Affiliations:** 1 Gerontopôle of Toulouse, Institute of Ageing, Toulouse UniversityHospital (CHU Toulouse), Toulouse, France; 2 Currently at the Northwest County Hospital, Murcia University, Murcia, Spain; 3 Toulouse UniversityHospital (CHU Toulouse), Toulouse, France.

**Keywords:** Depressive symptoms, magnetic resonance imaging, grey matter, white matter, diffusion tensor imaging

## Abstract

Depressive symptoms the most prevalent clinical condition in the field of mood disorders in older populations. Depressive symptoms are associated to poorer morbidity and mortality, and is considered a component of frailty and intrinsic capacity. Dementia could overlap with DS in clinical and brain abnormalities. Moreover, there are sex-differences in the field of Neuro- and Gero-science. To date, no review has addressed the neuro-anatomical basis of DS in older adults using magnetic resonance imaging (MRI), neither has investigated the discrimination of dementia nor sex-differences. This narrative review investigated studies about older adults; depressive symptoms evaluation via MRI, and published in English or Spanish over the past 7 years. Moreover, it evaluated dementia discrimination and sex-related differences. The most accurate evidence showed cerebral small vessel disease as a predictor of depressive symptoms worsening. Most studies were cross-sectional, with a coarse dementia screening and sex-unrepresentative samples. Cingulate cortex and hippocampus showed a negative association to depressive symptoms, and Precuneus cortex a positive association; although these inferences require further investigation. Additional research is needed to identify the brain imaging signature of depressive symptoms in older population (if any), and if this would be associated with sex and individuals’level of frailty and intrinsic capacity.

**Abbreviations:**
ACC: anterior cingulate cortex
AD: axial diffusivity
APOEε4: apolipoprotein Eε4
CA1: first region of hippocampal circuit
CC: cingulate cortex
CES-D: centre for epidemiologic studies depression scale
CSVD: cerebral small vessel disease
CT: cortical thickness
DS: depressive symptoms
DSp: depressive symptomatic participants
DTI: diffusion tensor imaging
FA: fractional anisotropy
GDS: geriatric depression scale
GM: grey matter
IC: intrinsic capacity
MD: mean diffusivity
MDD: major depressive disorder
MRI: magnetic resonance imaging
PCC: posterior cingulate cortex
RD: radial diffusivity
SS: somatic symptoms
TBSS: tract-based spatial statistics
TBV: total brain volume
WBA: whole-brain analysis
WM: white matter
WMH: white matter hyperintensity

## Introduction

At older ages, clinically relevant depressive symptoms (DS) frequently occur. The prevalence ranges from 10 to 14%, which for some cohorts represents 3-fold more than the observed for Major Depression Disorder (MDD) (1,2). DS are associated with similar functional and medical comorbidities of those found in MDD, and moreover, are considered its precursor (3). The impact of DS may be greater in older adults, compared to younger counterparts, due to their cumulative effect and normal age-related brain changes (4). They are proven to increase morbidity, disability and mortality risk (5,6). In addition, they represent the psychological domain of the intrinsic capacity concept (IC) (7) and have been linked to three IC domains (locomotion, cognition and sensory) (8,9). According to this evidence, appraising DS is a key point of the functional ability assessment in older populations, and suggests a more integrating and individualized health care model, which steps away from the obsolete disease-oriented medical care model (10).

In the past years, a growing body of literature has showed the neuro-anatomical basis of DS using magnetic resonance imaging (MRI). The areas which have received the most attention are those embedded in the neurobiological models of MDD. The frontal lobe, the hippocampus, and cingulate cortex (CC) are involved in the fronto-limbic network. The CC is divided into four functionally distinct regions: the anterior cingulate cortex (ACC), mid-cingulate cortex, posterior cingulate cortex (PCC), and retrosplenial cortex, which comprises the isthmus (11). Also the medial parietal cortex, referred as Precuneus, is involved in self-referential processing and episodic memory, and participates in the default mode network, whose hyperactivity is related to cognitive negative rumination process (12); all of which are elements of the proposed cognitive model of depression (13).

Another target of DS and brain-MRI studies, is white matter hyperintensities (WMH): hyper-intense areas, diffuse or patchy, in deep or periventricular distribution (i.e. in T2 MRI). Furthermore, white matter microstructure have been investigated through MRI-diffusion tensor imaging (DTI). DTI-MRI measures the diffusion of water molecules in neural fibers. Several DTI-indices can be measured, such as fractional anisotropy (FA); mean, radial, and axial diffusivity (MD, RD, AD). These measures can be locally assessed in pre-defined regions of interest, tracts of interest using tract-based spatial statistics (TBSS), or globally, with voxel-wise whole-brain analysis (WBA) (14).

These investigations help to understand the biological mechanisms of DS, and evidence suggest that they are similar to those found in MDD in older adults (15). Despite this, DS have received much less attention than MDD in literature. To our knowledge, no review has addressed the neural correlates of DS in older population yet. In addition to this, there is a broad evidence on how sex differences are found in the field of Neuro- and Gero-science (16,17). It is proved that there are sex-related differences in brain morphology (18,19) and that sex-related factors like hormones, stress or socio-economic status, differently affect the neurodevelopment (20-23). Moreover, in older populations, there is a high prevalence of concomitant depression and dementia (24). Both entities share similar changes, not only clinically, but also in brain morphology and dysfunction (25). Nevertheless, the clinical management, treatment and quality of life prognosis differ, underscoringthe importance of distinguishing these factors.

The current work aims at reviewing the main results of studies on MRI markers associated to DS in older adult populations. It also evaluates how sex-related differences and dementia discrimination are accounted.

## Method

This is a narrative review. We looked for original articles on (i) older population (≥62 years old), (ii) measuring DS, (iii) neuroimaging assessment through brain-MRI. Electronic searches were performed in January 2023 by one reviewer using different databases (eg: PubMed, Web of Science and Central, Epistemonikos, Tripdatabase). We focused on recent literature (i.e. past 7 years) published in English and Spanish. Studies related to a specific neurological disease, or other psychiatric disorders, or depression treatment distinct from antidepressants (i.e: electroconvulsive therapy) were excluded. We extracted information on study design, sample size (% female), mean age of subjects, cognitive performance screening, DS variable definition (dependent or independent, categorical or quantitative), DS scoring tool, MRI methods and focus, main outcomes and limitations.

## Results

**Figure 1:**
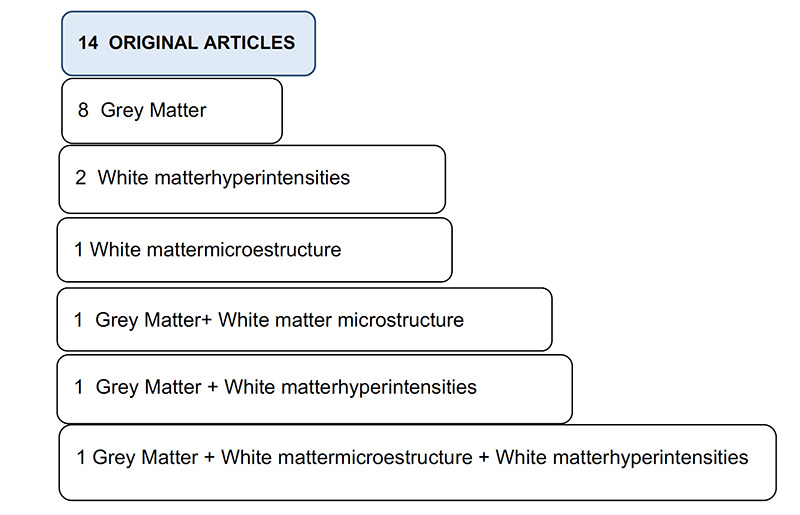
Summary of MRI-focus in selected studies

MRI studies have been focused on grey matter volume and thickness, and white matter abnormalities. Figure 1 shows the summary of main focus of the selected studies. Twelve of the 14 publications presented a cross-sectional design. Sample sizes ranged from 32 to 1,950 participants. Mean age of subjects ranged from 65 to 83 years and all were community-dwelling. The 2 cohort studies considered DS as an outcome variable, considering it a continuous variable (based on scoring tools).The most used DS scoring tool was the Center for Epidemiologic Studies Depression Scale (CES-D). Studies evaluating MRI multimodal markers were classified into 2 main sections: 1) Grey matter (GM) studies, 2) White matter (WM) studies. All studies were adjusted by age, sex and educational level; and some of them by cognitive performance tests. GM ones were also adjusted by total intracranial volume and WM ones by cardiovascular risk factors. Characteristics of key studies are shown in Tables 1 and 2.

**Table 1:** Descriptive characteristics of grey matter and white matter hyperintensities MRI studies

Authors	Study design	Subjects				Depressive symptoms		MRI Method and focus	Global results*	Limitations
		Sample size (%female)	Meanage (years- SD)	Cognitive Assessment	AD	Variable definition	Scoringtool			
Kumar et al., 2015	Cross-sectional Case-control	32 (57.6%)	76.2 (7.5)	Dementia excluded but assessment not outlined	Not accounted	Categorical	Minor depression= (low mood +/- loss of interest in activities) + (>1 item from DSM) for a month	1.5 T Volumetric	Minor depression had decreased **CT in the right Isthmus Cingulate;**compared to healthy counterparts, but did not correlate with symptoms severity or age.	Cross- sectional Sample size No confounders
Sloten et al., 2015	Longitudinal Cohort (4y)	1949 (56.6%)	74.6 (4.6)	Excluded by clinical interview based on DSMIV	Covariate	Dependent Independent Continuous Categorical	GDS >6 or new intake of AD = Incident Depressive Symptoms; (IDS)	1.5 T Cerebral Small Vessel Disease features (CSVD)	At baseline, higher number of subcortical infarcts and **lower total** **brain volume**were associated with higher IDS. Increase in **WMH**; incident **sub-cortical infarcts and** **Virchow-Robin spaces** and a decrease **in total** **brain volume**over time were associated with higher IDS. This association was stronger **in the deep brain** region and **Frontal.**GDS baseline was not associated to CSVD markers over time.	
Chang et al., 2015	Longitudinal Cohort(2y)	707 (63%)	71.3 (7.8)	MMSE (<20)	Not accounted	Dependent Continuous	GDS	WMH	**Severe deep WMH in** **APOE4 carriers**predicted progression of depressive symptoms, compared to mild WMH. This was not seen for APOE ε4 non-carriers	Not specific lobar allocation for WMH. Cognitive assessment Not AD as a covariate
Mclaren et al., 2016	Cross-sectional	41 (71%)	69.68 (6.8)	TICS	Not accounted	Continuous	CES-D Subscales: somatic symptoms, depressed mood, lack of affect.	3 T Volumetric	Higher score on depressed mood subscale was associated with larger GMV **in the left Posterior** **Cingulate Cortex**and reduced GMV in the **right** **Isthmus of the Cingulate** Higher score on the somatic symptoms subscale was associated with smaller GMV in **left** **Posterior Cingulate Cortex** No association for **Anterior Cingulate Cortex**	Cross- sectional Sample size Potential sex bias Cognitive assessment No AD as covariate
Szymkowicz et al., 2016	Cross-sectional	43 (69.7%)	68.9 (7)	TICS	Not significant covariate	Continuous	CES-D	3 T Volumetric	Higher CES-D score was associated with increased CT in the **right isthmus** **cingulate,** the bilateral middle **frontal gyrus** and increased CT in **the left** **precuneus**	Cross-sectional Sample size Potential sex bias
Zhou et al., 2016	Cross-sectional Case- control	36 (86%)	66.5 (4)	MMSE (<24)	Not accounted	Continuous Categorical (for description)	CES-D ≥8= DSp	3 T Volumetric	DSp had reduced GMV of **right parahippocampus**compared to healthy control. CES-D score was negatively correlated with GMV of **the left parahippocampus**, the **right hippocampus/ parahippocampus.**	Cross- sectional Sample size Potential sex bias No AD as a covariate
Szymkowicz et al., 2017	Cross-sectional	43 (69.7%)	68.9 (7.2)	TICS MMSE	Not significant covariate, not included	Continuous	CES-D	3 T Volumetric	CES-D scores counteract age-related volumetric decreases in the Subiculum and the CA1 of right **hippocampus.**	Cross-sectional Sample size Potential sex bias Cognitive assessment No AD as a covariate
Pink et al, 2017	Cross-sectional	1507 (50%)	77(4)	Multidisciplinar Assessment	Covariate	Continuous	BDI-II	3 T Volumetric	**Global CT**is negatively correlated to DS. **Frontal** **CT**was negatively correlated to DS; globally and in regions of the prefrontal Cortex, as: Dorso-lateral-prefrontal cortex, Ventro-lateral-prefrontal cortex, Orbito-frontal cortex. **Temporal CT**was negatively associated to DS; globally and in the Entorhinal Cortex. No association with **anterior cingulate cortex.** No association with **para-** and **hippocampus**after adjustment	Cross-sectional
Szymkowicz et al., 2018	Cross-sectional	77 (62%)	71.8 (10.6)	MOCA (<20)	Not significantcovariate	Independent Continuous	BDI-II subscale SomaticSymptoms	3T Volumetric	Somatic symptoms positively associated to **right Precuneus CT**(PCT). **PCT**not associated to age but the combination of age and increased somatic symptoms was negatively associated in left PCT.	Cross-sectional Sample size Potential sex bias Cognitive assessment
Szymkowicz et al., 2019	Cross-sectional	80 (40%)	70 (10.57)	MOCA (<20)	Not significant covariate	Continuous	BDI-II	3T Volumetric	Total DS and somatic symptoms (separetly) were associated with smaller **hippocampal volume.** Somatic symptoms were negatively associated with **Posterior Cingulate** **Cortex.**	Cross-sectional Sample size Cognitive assessment
Zeki et al., 2018	Cross-sectional	1111 (58%)	71 (9)	Multidomains Cognitive Assessment	Covariate	Categorical Independent	CES-D ≥16 = greater DS	1.5 T Volumetric WMH	Greater DS was associated with smaller **cerebral** **parenchymal fraction.** Greater DS was associated with greater odds of **subclinical brain infarcts.** No associations between greater DS and **WMH** **volume or hippocampal** **volume.**	Cross-sectional

AD= Antidepressant, APOE4= Apolipoprotein E4, BDI-II= Beck Depression Inventory 2ndedition,CES-D= Center for Epidemiologic Depression, CSVD= Cerebral Small Vessel Disease, CT= Cortical Thickness, DS= Depressive Symptoms, DS-p= Depressive Symptoms participants, DSM IV= Diagnostic and Statistical Manual of Mental Disorders 4rd edition, GDS= Geriatric Depression Scale, GMV = Grey Matter Volume; IDS= Incident Depressive Symptoms, MMSE= MiniMental State Examination; MOCA=Montreal Cognitive Assessment; 3MS= Modified MiniMental State, MRI= Magnetic Resonance Imaging, PCT= Precuneus Cortical Thickness, SD= Standard Deviation, T= Tesla; TICS= Telephone Interview for Cognitive Status, WMH= White Matter Hyperintensity, y=year. *: all shown results are statistically significant. **: defined by two of these circumstances: unexplained unequal sex-distribution sample, lack of sex based analysis, lack of sex bias discussion. Bold words: neural-structure of interest.

**Table 2:** Descriptive characteristics of diffusion tensor imaging MRI studies

Authors	Study design	Sample				Depressive symptims		MRI method and focus	Global results*	Limitations
		Sample size (%female)	Meanage (years- SD)	Cognitive Assessment	AD	Variable definition	Scoringtool			
Tudorascu et al., 2015	Cross-sectional Case-control	279 (44%)	83(3.3)	3 MS	Not accounted	Categorical	CES-D >10 = DSp	3 T Volumetric DTI WMH	DSp was associated with lower global **GMV** (more significantly in the left insula and anterior cingulate cortex), higher **WMH burden** and lower **FA** compared with non DSp.	Cross-sectional No AD as a covariate
Uden et al., 2015	Cross-sectional	438 (45.4%) History of Cerebral Vessel Disease (CVD)	65.1 (8.8)	Multiple Screening tool	Covariate	Categorical	CES-D >16/ use of AD = DSp	1.5 T DTI	WMH were periventri- cular, specially in frontal regions **Overall White Matter Skeleton** had lower **FA** and higher **MD** and **RD** in DSp compared to non-DSp. **Regionally:** The genu and the body of the corpus callosum, bilateral inferior fronto-occipital fasciculus, uncinate fasciculus, and corona radiata showed lower FA and higher: MD, AD and RD, in DSp compared to non-DSp. After adjustment for CVD these findings disappeared for FA and RD, but MD remained higher in the genu and body of corpus callosum, right anterior cingulum bundle, and bilateral superior corona radiate; and AD remained higher in the right superior corona radiata and right superior longitudinal fasciculus.	Cross-sectional Heterogeneous range of age All participants had vascular disease
Allan et al., 2016	Cross-sectional Case- control	358 (17%) Commun.	69(5)	MOCA	Excluded if used	Continuous Categorical	CES-D >10= DSp	3 T DTI Volumetric	DSp were associated with reductions **FA** and increases in **AD** and **RD** particularly in corpus callosum inferior and superior longitudinal fasciculi No relationships between DS (continuous) and GMV No association in between Framinghan Score Risk profile and DS.	Cross-sectional Potential sex bias**

*: all shown results are statistically significant. Bold words: neural - structure of interest. **: defined by two of these circumstances: unexplained unequal sex-distribution sample, lack of sex based analysis, lack of sex bias discussion (inspired by SAGER guidelines); AnD= antidepressant, AD= Axial Diffusivity; CD:=Cohen ‘s de, CES-D= Center for Epidemiologic Depression, CVD= cerebral vessel disease, DS= depressive symptoms, DSp= depressive symptoms participants, DTI= diffusion tensor imaging, FA= Fractional Anisotropy, GMV = Grey Matter Volume, MD= Mean Diffusivity, MOCA=Montreal Cognitive Assessment, MRI= Magnetic Resonance Imaging, 3MS= Modified Mini Mental State, RD= Radial Diffusivity; T= Tesla, WM= White Matter, WMH= White Matter Hyperintensity.

### Grey matter studies

Five broad anatomical categories can be distinguished in GM studies.

#### Frontal lobe

A cross sectional study reported that DS were negatively associated to the frontal cortex thickness, globally, and in regions of the prefrontal cortex: dorso-lateral prefrontal cortex, ventro-lateral prefrontal cortex and orbito-frontal cortex(26). In contrast, another study found a positive cross-sectional relationship between DS and thickness in the middle frontal gyrus (27).

#### Temporal Lobe

A correlation was found for DS and temporal cortex thinning and this was also observed in the Entorhinal cortex (26).Two studies, through two different scoring tools, found that increased DS were negatively associated with hippocampal volume (28,29). Moreover this was also found for the left and right parahippocampus (29). In addition, when subjects were categorized as depressive symptomatic participants (DSp), they presented lower hippocampal volume compared to healthy controls (29). However, this study did not account for antidepressant intake. Conversely, two studies found no association between hippocampal volume and DS. One conceptualized DS as “greater DS” (CES-D>16) (30) and the second lost the statistical significance after adjustment by antidepressant medication, comorbidities and global cognition (26). Furthermore, one study found that age lowering-effects on hippocampal volume were counteracted by DS in CA1 (first region of hippocampal circuit) and in the subiculum, in the right hemisphere (4).

#### Parietal Lobe

In the parietal lobe, only the precuneus has been studied in relation to DS.A cross-sectional study observed that more somatic symptoms (SS) (a DS subscale) were associated with greater right precuneus cortical thickness, nonetheless, when testing age and DS-subscales interaction, found that younger ages were associated with greater cortical thickness, whilst older ages were associated with less cortical thickness (31). Moreover, other study, in an exploratory analysis, showed greater thickness in the left precuneus with higher number of DS (27).

#### Cingulate Cortex

The ACC showed a negative association with DS in one study (32) whereas others did not find any (26,33). Larger left PCC volume was associated with higher scores on the depressed mood subscale, but higher scores on the SS subscale were associated to smaller volumes (28,33).The isthmus was the most addressed structure. Results were restricted to the right hemisphere, and showed the clearest findings with 2 studies supporting smaller volumes related to higher DS. One study found association between isthmus cortical thinning and minor depression (self-reporting low mood and/or loss of interest in activities and at least one additional Diagnostic and Statistical Manual of Mental Disorders V item, for one month of duration) compared to healthy controls (34). Other study found that higher scores on the depressed mood CES-D subscale were associated with smaller volumes of the right isthmus (33). Nevertheless, one study showed a positive association for DS and thickness in the right isthmus (27).

#### Global measures

##### Cortical thickness (CT)

CT has been found to be a better indicator of brain abnormalities related to DS than surface area (31). Nonetheless, only one study found that its thinning was correlated to DS burden (26).

##### Total Brain Volume (TBV) and Cerebral Parenchymal Fraction

One study, which presumed lower TBV as a marker of cerebral small vessel disease (CSVD), found that a decrease in TBV over time was associated with higher incident DS (35). Smaller cerebral parenchymal fraction was associated with greater DS even after adjustment for sociodemographic, behavioural, vascular risk factors, and antidepressant medication (30).

All associations investigated in every grey matter study are depicted in Table 3.

**Table 3:** Number of studies which support each association direction for every neuro-structure investigated

	Association direction		
Neuro-structure of Interest	Negative	Positive	None
Frontal lobe	1	1	
Temporal lobe	1		
Hippocampus	4	1	2
Entorhinal cortex	1		
Parietal lobe			
Precuneus		2	
Cingulate Cortex			
Anterior CC	1		2
Posterior CC	2	1	
Isthmus	2	1	
Global Cortical Thickness	1		
Brain volume	1		
Parenchymal Fraction	1		
TOTAL	15	6	4

### White matter studies

White matter studies could be classified into two main categories according to a macro or micro-scope.

#### Markers of cerebral small vessels disease (macro-scope)

One longitudinal study found that, baseline and incident subcortical infarcts, incident Virchow-Robin spaces and increased WMH volume over time, were associated to incident DS (Geriatric Depression Scale – GDS score >6). This association was stronger in subcortical areas and frontal region (36). Other study did not find an association between WMH volume and “greater DS” (CES-D ≥16) but found association for “greater DS” and greater odds of subclinical brain infarcts in models adjusted for vascular risk factors (30).

Interestingly, one longitudinal study based on the evidence of non-proportional association for WMH volume and DS, investigated the modification effect of apolipoprotein Eε4 (APOEε4) genotype in this context. Authors found that severe deep WMH (i.e. according to size of cap and band) among homo- and heterozygote APOEε4 carriers predicted progression of DS, compared to mild WMH, and this was not seen among APOEε4 non-carriers (36). On the other hand, other study did not find an association between APOEε4 and DS in the absence of WMH (26).

#### White Matter Microstructure

Regarding a micro-scope, three studies investigated MRI-DTI parameters. When focusing on overall measures (WBA), lower FA was associated to DSp (38), even when WMH were excluded (32). Also, higher MD and RD were observed among DSp (38). These results were associated to DS independently of age, sex, cognitive performance, and level of education (32,37). TBSS showed that DSp compared to non-DSp had lower FA and higher RD, MD and AD in the genu and the body of the corpus callosum, bilateral inferior fronto-occipital fasciculus, uncinated fasciculus, and corona radiate; higher MD in the right anterior cingulum bundle; and higher AD in the right superior longitudinal fasciculus (38). After adjusting for cognitive test, FA findings disappeared, and after adjusting for WMH and lacunar infarcts, FA and RD findings disappeared; results for MD and AD remained significant (38).Other study showed that DSp had reduced FA and increased AD and RD within the corpus callosum and the inferior and superior longitudinal fascicule; all 3 parameters remained significant after controlling by Framinghan Score Risk (cardiovascular risk tool assessment) (39).

## Discussion

Cerebral small vessels disease markers showed the clearest evidence. Higher number of subclinical infarcts, subcortical infarcts and white matter hyperintensities are associated to more depressive symptoms and predicted its progression. Interestingly, one study found that for APOEε4 genotype carriers, severe WMH was a predictor of DS worsening (36), but this was not seen in the absence of WMH (26), somehow suggesting that APOEε4 genotype must be accompanied by a pathological subtract for such an effect. These findings underscore the importance of medical management of CSVD for treatment and prevention of DS.

DTI studies also found consistent results: lower fractional anisotropy; and higher mean, radial, and axial diffusivity, overall, and in specific tracts, were associated to greater DS. To note, DTI changes can only be interpreted as damage in the WM micro-structure in the context of aging and disease-related studies, thus it cannot be attributed to a particular aspect of the brain micro-structure (e.g: myelinisation) (39). Additionally, a TBSS study, found that the decreased FA disappeared after adjusting for cognitive test, and after adjusting for WMH and lacunar infarcts, but results for MD and AD remained significant (37). FA combines contributions from different compartments of WM into a single metric, so it is believed to be the most sensitive and least specific DTI-index, possibly involved in CSVD and/or brain pathways for cognition. However, MD and AD could mirror other factors, which might be more specific of WM deterioration in older adults with DS. Another study found that reduced FA remained significant after controlling by Framingham Score Risk (cardiovascular risk tool assessment) what suggests that FA is sensitive to CSVD but not yet to cardiovascular risk factors.

Regarding grey matter studies, the majority found reduced volumes, thickness and surface associated with depressive symptoms. The most examined structures were the cingulate cortex and the hippocampus volume. This seems expected, since the CC and hippocampus are part of the fronto-limbic networks, nevertheless the frontal lobe has been scarcely studied and conclusions are inconsistent. Most of findings showed decreased hippocampal volume associated to greater DS. Nonetheless one study found that age lowering-effects on hippocampal volume were counteracted by DS in the hippocampus (4); what assumes an underlying enlarging effect of DS in hippocampal regions. These heterogeneous results might be explained by the timing in which the neurotoxic effects mediated through the hypothalamus-pituitary-adrenal axis affect the neural system (40). Similarly, the CC findings, mainly showed decreased cortical thickness associated to higher DS burden; however, some studies reported DS-related increasing effects (27,33). These divergent morphometric changes could be explained under the assumption of the differential DS effect along their course. There is evidence that the early stages of depression are associated to increased metabolic activity, blood flow, and inflammation (41), which leads to neurogenesis in the implicated areas and result in increased brain volumes; and this, could be presumably reverted over time, lowering the affected areas in later stages (42). Another investigated area was the Precuneus. Despite being involved in an array of higher-order cognitive functions, only 2 studies focused on it. They, consistently, found that, increased Precuneus cortex is related to higher DS.

These neural correlations should not endorse a discrete and organic health scope which favours diagnosis, treatment, and disease monitoring, based on complementary tests. On the contrary, they herald the embracement of cognitive-neural-affective dimensions, and thereby, the importance of DS as a proxy of clinical global health. Furthermore, these results stand against those who argue that DS is an age-related condition. Not only because of the proven evidence of its association to brain abnormalities independently of age, but also to the fact that changes identified in both conditions are different (43).

Due to the heterogeneity of the methodology studies, we acknowledge some limitations when making conclusions out of the selected studies. These challenges deserve further commentaries.

### Challenges

#### Study design

The majority of studies have been limited to a cross-sectional design. Cross-sectional studies are particularly affected by uncertain theoretical basis of causality (44), what is reflected here, since there is no clear evidence that could, chronologically, clarify the neurobiology of DS in older people. This affects at least 3 of the outstanding questions arisen from this review: causality direction, morphometric changes direction and exclusion of dementia. How DS and brain changes relate to each other in terms of temporal sequence is not homogeneously understood. Often, periods of heightened stress precede depression. Stress triggers glucocorticoids secretion and this has effects in the brain through the hypothalamus-pituitary-adrenal axis (45). In this context, the neurotoxicity hypothesis entails that prolonged exposure to glucocorticoids reduces the ability of neurons to resist insults, what would lead to brain abnormalities as an end product of years of exposure (46). Moreover, there is evidence that depression leads to an increased risk of inflammatory response and unhealthy lifestyle patterns, which raises the cardio and cerebral-vascular risk (47). Accordingly, it was shown that increased exposure to stressful life events among depressed older people was associated with increases in WMH volume over a 2-year period (48). In contrast, the vulnerability (49) and vascular depression hypotheses (50) postulate the opposite. They suggest that abnormalities in GM volume and WM integrity, respectively, would increase the vulnerability to present DS. In this framework, the only two longitudinal studies considered DS as an outcome of brain damage (35,36). Furthermore, these hypotheses are not mutually exclusive from a developmental perspective. The relationship between DS and brain abnormalities could not simply be linear, but turned into a reversible feedback which hinders the comprehension. In line with this, one longitudinal study aimed at testing the reverse causality hypothesis (36) and found that, whereas CSVD baseline and progression over time was associated to DS, DS at baseline were not associated to CSVD progression, however, they did not test if DS changes would affect CSVD markers of progression.

#### Dementia discrimination

Dementia and DS discrimination is crucial, given that both share clinical and brain abnormalities. A cross-sectional study showed that the differences found for FA in DSp compared to non-DSp disappeared after adjustment for a cognitive index (38). Factors influencing the appropriateness of dementia discrimination comprises: cognitive assessment tools, the threshold chosen, its consideration in the statistical analysis and cognitive follow-up. Follow–up was not done by any of the selected studies. One study did not specify the method for cognitive assessment (Kumar et al., 2015), while others conducted a telephonic interview (4,27,33). Seven studies used validated screening tools on-site (28,29,31,32,35,36, 38). Lastly, 3 studies conducted a multidisciplinary assessment which could discriminate mild cognitive impairment. Six studies had cognitive-performance-test into account for the statistical adjustment (26,29,32,36,37,38), while other three, which included patients with different level of cognitive status, did not (4,27,31).One study with cognitively normal older participants showed that, after a mean follow-up of 5 years, the CSVD findings caused DS independently of the cognitive function at baseline (36). In order to conclude how DS relate to dementia or not, efforts should be put on studies which evaluate cognitive function with specific tools, monitor cognitive performance overtime, and account for it in statistical analysis.

#### Sex-related bias

After applying the guidelines for reporting of Sex and Gender Equity from the European Association of Science Editors (52), we found several caveats to be noted. Ten studies used the term “sex”,3used “gender” (29,32,34), an one used both terms interchangeably (33). Using the term “gender”, when referring to male or female, leads to a bio-psycho-social misconception. These terms have different meanings. Sex is a biological attribute, whereas gender is a socially constructed role (53). Six studies had samples in which one of both sexes represents less than 40% of the total: male participants were underrepresented being 37%, 29%, 30.3%, 30.3%, 37% and 14%of the sample (27,28,29,31,36) , and in another study, female counted for 14% (39). This raises the risk of sex-bias, when conclusions are to be implemented to general population. As an example, some of these men-underrepresented studies found a positive association for DS and brain volumes. Having into account the proven dimorphic brain organization by sex, as increased cortical thickness in female brain (18), these inferences are likely to be affected by sex-related bias. Nevertheless, only one study discussed it as a generalizability issue (39). For all studies, sex was included as a confounder, which demonstrates its consideration as an explanatory variable, but surprisingly, only two studies accounted for it as a modifier effect, showing that sex could modify the relationship between DS and GMV. One study found that sex interaction for GMV was significant, whereas for WMH and FA it was not (32); and the other did not find significant interaction for sex and “greater DS” (30). None of the studies stratified results by sex. These oversights limit the external validity of research findings and their applicability to clinical practice.

#### Depressive symptoms screening tool

Another concern is the lack of consensus on how to measure DS. There are diverse screening tools and different cut-offs to categorize participants, thus it is difficult to generalize findings (see table 1 and 2). The CES-D was the most used scale, and the cut-offs varied (8 or 10 or 16). The GDS and the Beck Depression Inventory were also used, while other studies used participant self-administered questionnaires and others, structured clinical interviews. Consequently, correlating each screening tool and agreeing on their cut-off is highly needed. On the other hand, this might encourage other investigations, in the field of brain imaging and health conditions, to consider DS as a variable to account for.

#### Antidepressant drugs

Some studies considered the prescription of antidepressants as a synonymous of presence of DS(38), what risks to be a misconception, since the so-called antidepressants are also indicated for other pathologies (e.g. insomnia, anxiety or neuropathic pain), might be inadequately prescribed (54), or participants taking them are highly likely to have been diagnosed with MDD. In addition, antidepressants could influence brain imaging findings (55).

## Conclusions

This review concludes that the most reliable evidence shows cerebral small vessel disease as a predictor of DS worsening. Also, cross-sectional associations related: lower FA, higher RD, higher AD, thinner frontal and temporal cortex, lower hippocampal volume, and increased Precuneus thickness to higher DS. Results related to cingulate cortex, are still controversial and demand further investigation. Dementia discrimination was inaccurate in some of studies and none of them followed it up over time. Moreover, sex-related differences were mostly overlooked.

Taking into account that MDD is the most common psychiatric disorder seen in community-dwelling older adults, and that DS largely outnumbers it, DS in older people deserve research efforts from the translational standpoint. Moreover, disentangling the interdependent links in between DS and the intrinsic capacity domains is an underpinning pathway for achieving an integrated care for older people (56), which focuses on preserving “the health related attributes that enable people to do and to be what they have a reason to value” (57). In light of this review, longitudinal studies, investigating DS on older adults and its association to neurobiological markers, mainly: cingulate, frontal and precuneus cortex, and hippocampal volume, are needed. Moreover, accurately discarding the influences of cognitive impairment, nervous system drugs, and addressing sex-differences, would build a robust evidence in this field of Neuro and Geroscience.
